# Arthroscopy versus mini-arthrotomy approach for matrix-induced autologous chondrocyte implantation in the knee: a systematic review

**DOI:** 10.1186/s10195-021-00588-6

**Published:** 2021-06-16

**Authors:** Filippo Migliorini, Jörg Eschweiler, Filippo Spiezia, Bryan J. M. van de Wall, Matthias Knobe, Markus Tingart, Nicola Maffulli

**Affiliations:** 1 Department of Orthopaedic and Trauma Surgery, RWTH University Hospital Aachen, Pauwelsstraße 30, 52074 Aachen, Germany; 2 Department of Medicine, Surgery and Dentistry, University of Salerno, Via S. Allende, 84081 Baronissi, SA Italy; 3 School of Pharmacy and Bioengineering, Keele University School of Medicine, Thornburrow Drive, Stoke on Trent, England; 4 Barts and the London School of Medicine and Dentistry, Centre for Sports and Exercise Medicine, Mile End Hospital, Queen Mary University of London, 275 Bancroft Road, London, E1 4DG England; 5 Department of Orthopaedic and Trauma Surgery, Ospedale San Carlo Potenza, Potenza, Italy; 6 Department of Orthopaedic and Trauma Surgery, Lucerne Cantonal Hospital, Lucerne, Switzerland

**Keywords:** Knee, Chondral defect, Autologous chondrocyte implantation, mACI, Arthroscopy, Mini-arthrotomy

## Abstract

**Background:**

Matrix-induced autologous chondrocyte implantation (mACI) can be performed in a full arthroscopic or mini-open fashion. A systematic review was conducted to investigate whether arthroscopy provides better surgical outcomes compared with the mini-open approach for mACI in the knee at midterm follow-up.

**Methods:**

This systematic review was conducted following the PRISMA guidelines. The literature search was performed in May 2021. All the prospective studies reporting outcomes after mACI chondral defects of the knee were accessed. Only studies that clearly stated the surgical approach (arthroscopic or mini-open) were included. Only studies reporting a follow-up longer than 12 months were eligible. Studies reporting data from combined surgeries were not eligible, nor were those combining mACI with less committed cells (e.g., mesenchymal stem cells).

**Results:**

Sixteen studies were included, and 770 patients were retrieved: 421 in the arthroscopy group, 349 in the mini-open. The mean follow-up was 44.3 (12–60) months. No difference between the two groups was found in terms of mean duration of symptoms, age, body mass index (BMI), gender, defect size (*P* > 0.1). No difference was found in terms of Tegner Score (*P* = 0.3), Lysholm Score (*P* = 0.2), and International Knee Documentation Committee (IKDC) Score (*P* = 0.1). No difference was found in the rate of failures (*P* = 0.2) and revisions (*P* = 0.06).

**Conclusion:**

Arthroscopy and mini-arthrotomy approaches for mACI in knee achieve similar outcomes at midterm follow-up.

**Level of evidence:**

II, systematic review of prospective studies.

## Introduction

Focal chondral defects of the knee are common [1]. Hyaline cartilage is avascular, alymphatic, and hypocellular, with low metabolic activity [2–4]. Given these proprieties, the healing process often does not result in restitutio ad integrum, and residual defects are common [5, 6]. Symptomatic chondral defects are debilitating, and may lead to retirement from sports activities [7]. In patients with focal chondral defects, surgical treatment is often required [8, 9]. For smaller defects, microfractures are commonly performed [10–14]. Matrix-induced autologous chondrocyte implantation (mACI) has been commonly used to address bigger defects [15, 16]. During mACI, chondrocytes are harvested from a nonweightbearing zone of the articular cartilage of the knee in a first surgical session, seeded over a membrane, then expanded in vitro [17, 18]. In a second surgical session, the membrane loaded with autologous expanded chondrocytes is trimmed to fit the defect size, then placed into the defect [19, 20]. This second surgical session can be performed arthroscopically or with an arthrotomy in a minimally invasive fashion (mini-open). Whether the surgical approach influences the surgical outcome of mACI in the knee has not been previously investigated. Thus, a systematic review was conducted to investigate whether arthroscopy provides better surgical outcomes compared with the mini-open approach for mACI in knee at midterm follow-up. The focus of the present work was on patient-reported outcome measures (PROMs) and complications.

## Material and methods

### Search strategy

This systematic review was conducted following the Preferred Reporting Items for Systematic Reviews and Meta-Analyses (PRISMA) guidelines [21]. The PICOTD framework was followed:

- P (Problem): knee chondral defect;
- I (Intervention): mACI;
- C (Comparison): arthroscopy versus mini-open surgery;
- O (Outcomes): clinical scores and complications;
- T (Timing): $\ge$ 12 months follow-up;
- D (Design): prospective trials.

### Data source and extraction

Two authors (**;**) independently conducted the literature search in January 2021. The main online databases were accessed: PubMed, Google scholar, Embase, and Scopus. The following keywords were used in combination: *chodral, cartilage, articular, damage, defect, injury, chondropathy, knee, pain, matrix-induced, autologous, chondrocyte, transplantation, implantation, mACI, therapy, management, surgery, arthroscopy, mini-open, outcomes.* The same authors performed separately the initial screening. The full text of the articles of interest was accessed. A cross reference of the bibliographies was also conducted. Disagreements were debated and solved by a third author (**).

### Eligibility criteria

All the studies reporting outcomes after mACI for knee chondral defects were accessed. According to the authors’ language capabilities, articles in English, German, Italian, French, and Spanish were eligible. Only prospective studies with levels I to II of evidence, according to Oxford Centre of Evidence-Based Medicine [22], were considered. Only studies that clearly stated the fashion of the surgical approach (arthroscopic or mini-open) were included. Procedures other than mACI were excluded. Only studies reporting a follow-up $\ge$ 12 months were considered eligible. Animal or in vitro studies were not eligible. Studies investigating other surgical approaches rather than arthroscopic or mini-open were not eligible. Studies reporting data from combined surgeries were not eligible. Studies combining mACI with other less committed cells (e.g., mesenchymal stem cells) were not considered. Reviews, comments, letters, editorials, and techniques were not eligible. Only articles reporting quantitative data under the outcomes of interest were considered for inclusion. Missing data under the outcomes of interest warranted exclusion from the present study. Table 1 displays the eligibility criteria.

**Table 1:** Eligibility criteria

Eligibility criteria
Inclusion criteria
Clinical studies reporting outcomes following mACI for chondral defects of the knee
English, German, Italian, French, and Spanish languages
Prospective studies with level I to II of evidence [22]
Fashion of the surgical approach (arthroscopic or mini-open) clearly stated
Length of the follow-up $\ge$ 12 months
Report quantitative data under the outcomes of interest
Exclusion criteria
Animal or in vitro studies
Other surgical approaches rather than arthroscopic or mini-open
Studies reporting data on combined surgeries
Studies reporting data on non-mACI procedures
Studies enhancing mACI with other less committed cells
Reviews, comments, letters, editorials, and techniques studies

### Data extraction

Two independent authors (**;**) performed data extraction. Study generalities (author, year, journal, type of study) and patient baseline demographic information were collected (number of samples and related mean BMI and age, duration of the symptoms, duration of the follow-up, percentage of female). For every approach, the following data were retrieved: Lysholm Knee Scoring Scale [23], Tegner Activity Scale [24], and International Knee Documentation Committee (IKDC) [25] Score. Data from complications were also collected: rate of failures and revisions.

### Methodology quality assessment

The methodological quality assessment was performed by two independent authors (**;**). The risk of bias graph tool of the Review Manager Software (The Nordic Cochrane Collaboration, Copenhagen) was used. The following risks of bias were evaluated: selection, detection, attrition, and other source of bias.

### Statistical analysis

The statistical analyses were performed with IBM SPSS Version 25. Continuous data were reported as mean difference (MD) and standard deviation. For binary data, odds ratio (OR) effect measure was calculated. The confidence interval (CI) was set at 95% in all the comparisons. *t*-Test and $\chi$^2^ tests were evaluated for continuous and binary data, respectively, with *P* < 0.05 considered statistically significant.

## Results

### Search result

**Fig. 1:**
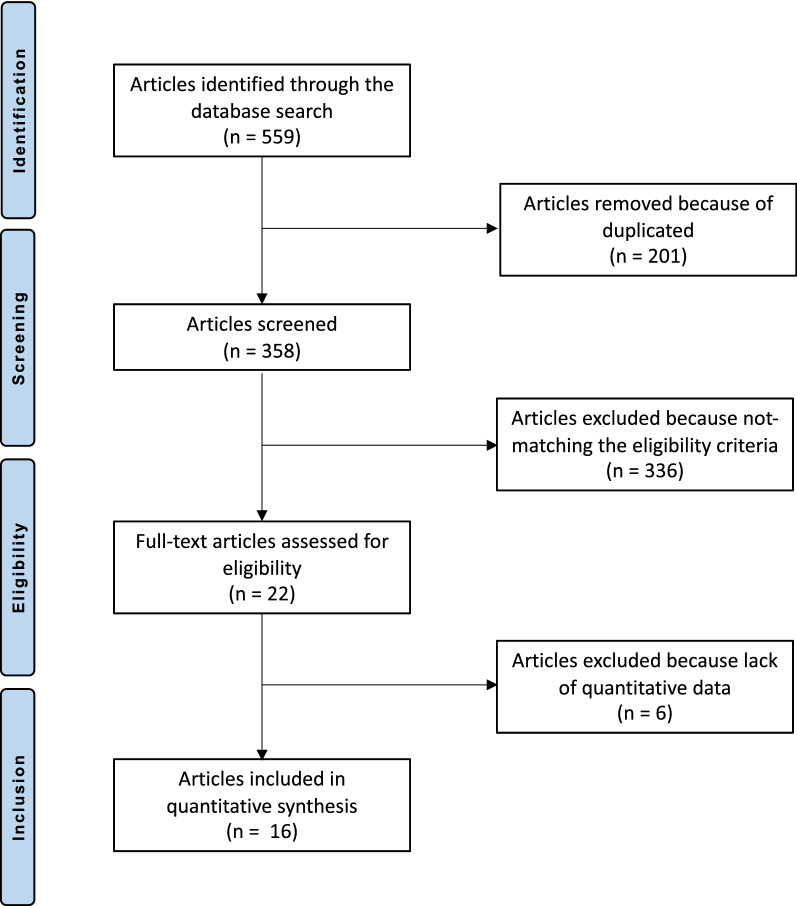
Flowchart of the literature search

The literature search identified 559 clinical investigations. Of them, 201 were excluded as they were duplicates. A further 342 articles were excluded because they did not fulfill our eligibility criteria: not focused on mACI (*N* = 171), not clearly stating the approach (*N* = 74), retrospective nature of the study design (*N* = 29), performing arthrotomy (*N* = 27), combined with stem cells (*N* = 13), other (*N* = 19), not reporting quantitative data under the outcomes of interest (*N* = 6), language limitations (*N* = 3). This left 16 studies for inclusion in the present investigation: six randomized controlled trials (RCTs) and ten non-RCTs. The flowchart of the literature search is shown in Fig. 1.

### Methodological quality assessment

**Fig. 2:**
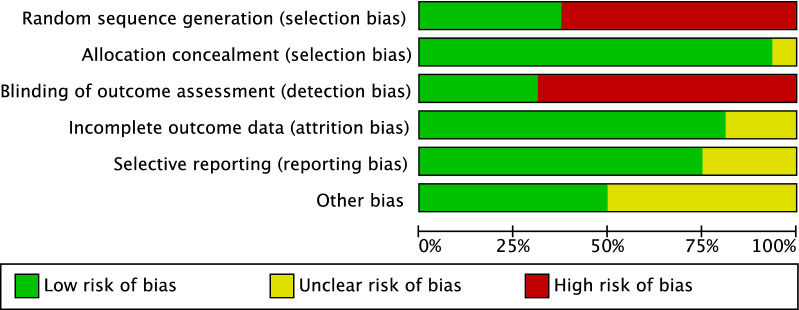
Methodological quality assessment

Given the limited number of included RCTs, the graph evidenced moderate risk of selection bias. The risk of selection bias of allocation concealment was low. The risk of detection bias was moderate, since the outcome assessments were rarely blinded. The risk of attrition and reporting bias were both moderate to low, as were the risk of other biases. Concluding, the overall review of the authors’ judgments about each risk of bias item was low, attesting to this study’s good methodological assessment. The risk of bias graph is shown in Fig. 2.

### Patient demographics

Data from 770 patients were retrieved: 421 in the arthroscopy group, 349 in the mini-open group. The mean duration of symptoms before the index surgery was 48.6 (26.4–91.2) months. Women accounted for 35% (273 of 770) of the sample. The mean age of the patients was 34.1 ± 4.6 years, and the mean BMI was 25.1 ± 0.8 kg/m^2^. The mean defect size was 4.0 ± 1.4 cm^2^. The mean follow-up was 44.3 (12–60) months. No difference between the two groups was found in terms of mean duration of symptoms, age, BMI, gender, or defect size (*P* > 0.1). Generalities and demographic of the studies are presented in Table 2.

**Table 2:** Generalities and patient baseline of the included studies

Author, year	Journal	Study design	Follow-up (months)	Approach	Patients	Female (*%*)	Mean age	Mean BMI	Mean size (cm^2^)
Akgun et al. 2015 [18]	*Arch Orthop Trauma Surg*	RCT	24	Control group	7	57	32.3	24.1	2.9
				Mini-arthrotomy	7	57	32.7	24.3	3
Basad et al. 2010 [17]	*Knee Surg Sports Traumatol Arthrosc*	RCT	24	Mini-arthrotomy	40	38	33	25.3	
				Control group	20	15	37.5	27.3	
Basad et al. 2015 [26]	*Knee Surg Sports Traumatol Arthrosc*	Non-RCT	60	Mini-arthrotomy	25	37	32	24	
Becher et al. 2017	*J Orthop Surg Res*	RCT	36	Arthroscopy	25	32	33	24.9	4.8
				Arthroscopy	25	16	34	25.6	4.9
				Arthroscopy	25	40	34	25.1	5.2
Ebert et al. 2012 [27]	*Arthroscopy*	Non-RCT	24	Arthroscopy	20	50	34	26.6	2.7
Ebert et al. 2016 [28]	*Am J Sports Med*	Non-RCT	60	Arthroscopy	31	51	35	26	2.52
Efe et al. 2012 [29]	*Am J Sports Med*	Non-RCT	24	Mini-arthrotomy	15	60	26		
Ferruzzi et al. 2008 [30]	*J Bone Joint Surg*	Non-RCT	60	Control group	48	38	32		6.4
				Arthroscopy	50	28	31		5.9
Filardo et al. 2011 [31]	*Am J Sports Med*	Non-RCT	84	Arthroscopy	62	23	28		2.5
Filardo et al. 2014 [32]	*Am J Sports Med*	Non-RCT	84	Arthroscopy	131	35	29	24	2.3
Kon el al. 2011 [33]	*Am J Sports Med*	Non-RCT	61	Arthroscopy	22	32	46	24.7	2.6
			58	Mini-arthrotomy	39	35	45	25.6	3.1
Marlovits et al. 2012 [34]	*Am J Sports Med*	Non-RCT	60	Mini-arthrotomy	24	12	35		5.1
Niemeyer et al. 2016 [35]	*Am J Sports Med*	RCT	12	Mini-arthrotomy	25	33	33	24.9	4.8
				Mini-arthrotomy	25	16	34	25.6	4.9
				Mini-arthrotomy	25	40	34	25.1	5.2
Niemeyer et al. 2019 [36]	*Orthop J Sports Med*	RCT	24	Mini-arthrotomy	52	36	36	25.7	2.2
				Control group	50	44	37	25.8	2
Saris et al. 2014 [37]	*Am J Sports Med*	RCT	24	Mini-arthrotomy	72	37	35	26.2	4.8
				Control group	72		33	26.4	
Siebold et al. 2018 [38]	*Knee Surg Sports Traumatol Arthrosc*	Non-RCT	34.8	Arthroscopy	30	36	36	23.8	6

### Efficacy of the procedure

At a mean follow-up of 44.3 (12–60) months, all PROMs of interest were improved (Table 3): VAS (MD −3.2; *P* = 0.008), Tegner (+2.2; *P* = 0.001), Lysholm (+31.9; *P* = 0.0002), IKDC (+33.2; *P* < 0.0001).

**Table 3:** Improvement of PROMs from baseline to last follow-up

Endpoint	Baseline	Last FU	MD	*P*
VAS	4.9 ± 1.3	1.8 ± 0.5	−3.2	0.008
Tegner	2.6 ± 1.4	4.8 ± 1.0	+2.2	0.001
Lysholm	50.1 ± 7.0	82.0 ± 8.9	+31.9	0.0002
IKDC	38.9 ± 9.0	72.1 ± 7.9	+33.2	< 0.0001

*FU* follow-up, *MD* mean difference

### Outcomes of interest

No difference was found in terms of Tegner Score (MD 0.5; *P* = 0.3), Lysholm Score (MD 6.9; *P* = 0.2), and IKDC Score (MD 6.8; *P* = 0.1). Similarly, no difference was found in the rate of failures (OR 1.4; *P* = 0.2) and revisions (OR 0.1; *P* = 0.06). Results of the scores are presented in Table 4 and those of complications in Table 5.

**Table 4:** Results of the scores

Endpoint	Arthroscopy (*N* = 412)	Mini-open (*n* = 349)	MD	*P*
Tegner	5.2 ± 0.2	4.8 ± 1.3	0.4	0.3
Lysholm	81.9 ± 5.9	88.7 ± 4.7	−6.9	0.2
IKDC	76.7 ± 6.6	70.0 ± 6.1	6.8	0.1

*MD* mean difference

**Table 5:** Results of the complications

Endpoint	95% CI	OR	*P*
Failure	0.79–2.57	1.4	0.2
Revision surgery	0.01–1.14	0.1	0.06

*OR* odds ratio

## Discussion

This systematic review was conducted to investigate whether arthroscopy provides better surgical outcomes compared with the mini-open approach for mACI in knee at midterm follow-up. According to the main findings of the present study, no difference was found between the two approaches in terms of PROMs. Additionally, at a mean of 44 months follow-up, no difference was found in the rate of failure and revision surgeries.

We were able to identify one study that compared open ACI covered by autologous periosteal flap (pACI) versus arthroscopic mACI [30]. PACI was performed in the fashion described by Bittermber et al. [39]. During arthroscopic mACI, expanded chondrocytes are seeded into a three-dimensional hyaluronic acid membrane (Hyaff-11; Fidia Advanced Biopolymers, Abano Terme, Italy), producing the scaffold called Hyalograft C. The membrane was then delivered into the defect in a “dry” arthroscopy after trimming using a suitable sized cylindrical cutting device. If necessary, several grafts can be applied to fill the defect, using an arthroscopic impactor. This technique produced better results compared with the open pACI in terms of IKDC and complications. However, whether this superiority arose from the approach or the surgical technique is unclear. The same arthroscopic technique was used by Filiardo et al., who reported very good results [31, 32]. The same arthroscopic technique using Hyalograft C was compared with a mini-open mACI (22 versus 39 patients, respectively) [33]. However, they used a porcine resorbable collagen I/III membrane for the mini-open procedure. They found comparable results at 5 years follow-up. There were four failures in the arthroscopic group (18.2%) and eight in the mini-open group (20.5%), with no statistically significant difference between them. The arthroscopic group reported a greater IKDC (55.9 ± 22.1 versus 67.4 ± 21.5; *P* = 0.05) at 1-year follow-up, with no differences at 24- and 60-month follow-up. These results suggested that the arthroscopic approach allows quicker recovery, but similar outcomes at midterm follow-up. However, they used two different membrane (Hyalograft C versus porcine resorbable collagen I/III membrane). Thus, it is unclear whether the quicker recovery seen at 12-month follow-up related to the approach or the different membrane. Two studies by Ebert et al. [27, 28] performed a similar full-arthroscopic procedure: after shaving and debridement, the defect was mapped in several planes using a graduated arthroscopy probe. The membrane was trimmed so as to be slightly oversized. At dry arthroscopy, the defect was dried using an arthroscopic sucker, and an adrenaline-soaked patty was pressed onto the subchondral bone to further dry it and prevent bleeding. Using a no-valves large-bore arthroscopic cannula, the membrane was positioned into the defect. The membrane was successively extracted out of the joint, and the size was finalized to correctly fit the defect ensuring correctly orientation of the graft. The membrane was glued, and a Silastic Foley catheter was introduced in the knee. The balloon was then inflated with saline for 30 s to compress the membrane and to secure it to the defect. There were minor variations in the execution of the surgical techniques in the arthroscopic group; minor variations were also evidenced in the mini-arthrotomy group. Indeed, two studies avoid membrane fixation [17, 29], while three studies [17, 26, 34, 37] employed fibrin glue to fix the graft. In one study, in addition to the glue, the membrane was sutured to ensure stability [18]. In the mini-open group, one study [29] used a resorbable collagen I graft, while five [17, 18, 26, 34, 37] used a resorbable collagen I/III membrane. Other studies enhanced the membrane with chondrocyte spheroids (Chondrosphere) [35, 36, 38]. Concluding, variations were evident both in the arthroscopic and mini-arthrotomy groups, and represent the most important limitations of the present study. We were unable to identify studies that compare arthroscopy and mini-open with the same protocol of membrane choice and fixation. Overall, the analyses were limited to a restricted number of procedures, representing another important limitation. The surgical techniques presented some minimal differences between authors, and cell culture and expansion protocols were heterogeneous between studies. Hence, these results should be been interpreted within the limitations of the present investigation, opening new perspectives and challenges to future studies.

## Conclusion

Arthroscopy and mini-open approaches for mACI in patients with chondral defects of the knee score were similar at midterm follow-up.

## Data Availability

The data underlying this article are available in the article and in its online supplementary material.

## Abbreviations

mACI: Matrix-induced autologous chondrocyte implantation
PROMs: Patient-reported outcome measures
RCT: Randomized clinical trial
BMI: Body mass index
IKDC: Knee Documentation Committee
MD: Mean difference
OR: Odds ratio
CI: Confidence interval
pACI: Periosteal-flap autologous chondrocyte implantation
